# Extraction and Characterization of Essential Discharge Patterns from Multisite Recordings of Spiking Ongoing Activity

**DOI:** 10.1371/journal.pone.0004299

**Published:** 2009-01-28

**Authors:** Riccardo Storchi, Gabriele E. M. Biella, Diego Liberati, Giuseppe Baselli

**Affiliations:** 1 Department of Biomedical Sciences, University of Modena, Modena, Italy; 2 Institute of Molecular Bioimaging and Physiology, Milan, Italy; 3 National Research Council, Institute of Molecular Bioimaging and Physiology, Milan, Italy; 4 Department of Electronic and Information, National Research Council, Politechnic School of Milan, Milan, Italy; 5 Department of Biomedical Engineering, Politechnic School of Milan, Milan, Italy; Indiana University, United States of America

## Abstract

**Background:**

Neural activation patterns proceed often by schemes or motifs distributed across the involved cortical networks. As neurons are correlated, the estimate of all possible dependencies quickly goes out of control. The complex nesting of different oscillation frequencies and their high non-stationariety further hamper any quantitative evaluation of spiking network activities. The problem is exacerbated by the intrinsic variability of neural patterns.

**Methodology/Principal Findings:**

Our technique introduces two important novelties and enables to insulate essential patterns on larger sets of spiking neurons and brain activity regimes. First, the sampling procedure over N units is based on a fixed spike number k in order to detect N-dimensional arrays (k-sequences), whose sum over all dimension is k. Then k-sequences variability is greatly reduced by a hierarchical separative clustering, that assigns large amounts of distinct k-sequences to few classes. Iterative separations are stopped when the dimension of each cluster comes to be smaller than a certain threshold. As threshold tuning critically impacts on the number of classes extracted, we developed an effective cost criterion to select the shortest possible description of our dataset. Finally we described three indexes (C,S,R) to evaluate the average pattern complexity, the structure of essential classes and their stability in time.

**Conclusions/Significance:**

We validated this algorithm with four kinds of surrogated activity, ranging from random to very regular patterned. Then we characterized a selection of ongoing activity recordings. By the S index we identified unstable, moderatly and strongly stable patterns while by the C and the R indices we evidenced their non-random structure. Our algorithm seems able to extract interesting and non-trivial spatial dynamics from multisource neuronal recordings of ongoing and potentially stimulated activity. Combined with time-frequency analysis of LFPs could provide a powerful multiscale approach linking population oscillations with multisite discharge patterns.

## Introduction

In the last twenty years, studies on information encoding in the nervous system have provided fundamental insights into the nature of neural inner dynamics and of sensorimotor representation and coding of the external world. Powerful and flexible statistical techniques have grown in time [1]–[5], improving the analyses of stimulus-response experimental paradigms along with their corollary complexities.

Scant attention has been paid to other dynamic features such as spontaneous or ongoing activity. Nevertheless, this feature represents 90% percent of the whole metabolic exertion of the brain [6]. An exhaustive description of ongoing activity as a kind of substrate intermingling with signals generated by external sources, could provide fundamental insights into nervous system dynamics.

Spontaneous neuronal population activities from many sites in the central nervous system present complex combinations of different oscillation frequencies [7]–[9]. Regular and repetitive motifs nest within the frames of these global rhythms. Furthermore, stereotyped patterns of specific neuron subsets have been evidenced in many different experimental conditions such as at the onset of UP states [10] or in *in vitro* recordings with calcium imaging [11], [12].

Because of the surging number of items, the statistical evaluation of all possible discharge patterns in multisite recordings of even a few channels quickly goes out of control. Nevertheless, pioneering studies and further advancements have developed algorithms capable of recognizing small, repetitive and well-timed patterns in tonic activity regimes [13]–[17]. Still, timing is likely not the only encoding means valid in the CNS.

Repetitive spontaneous and evoked neuronal activation patterns evidenced time warping features, namely duration independent activity episodes, in most diverse behavioral conditions [18]–[20]. These features may represent an additional piece of the multifarious presence of noise, a pervasive trait throughout the bottom-up (or top-down) scaling of brain analyses [21], that affects the immediate detection of accessible regular and repetitive patterns. In any case the crucial role of noise in brain operations, as it has become evident in recent years, has gone largely unknown and is a current subject of wide debate.

In the context of a lack of comfortable theoretical background for the present scenario, we tried to design an efficient algorithm to detect the presence of regular motifs in all ongoing activity regimes and for most variable numbers of spikes or recording traces. We then developed and evaluated a set of measures to characterize such motifs in terms of spatial structure, complexity and temporal evolution.

Our approach is conceptually different from previous works as we introduced several important novel features. First, in addition to constant time bin sampling, we introduced a conditional sampling based on keeping the number of spikes constant in each sample. This procedure allows the rejection of common mode frequency modulations. Second, we applied a clustering procedure in order to extract a reasonable number of classes from the wide variety of distinct patterns and to reduce the effect of noise. Then, following a cost criterion, we extracted a selection of the most frequently occurring classes that we called essential classes (EC). Finally, we developed three indexes, labeled *C*, *S* and *R*, evaluating respectively the average pattern complexity (C), the structure of the ECs (*S*) and their stability in time (*R*). We applied such indexes both to simulated and to real data. We mainly focused on the results obtained by using conditional sampling. However the results obtained with constant time bin sampling will be reported as well and commented for comparison.

In the Methods we describe the whole algorithm flow obtained by cascading constant time bin sampling or conditional sampling, the clustering procedure and the extraction of ECs (respectively subsections 1,2 and 3). We then describe the indexes *C*, *R* and *S* (subsection 4) and provide a detailed explanatory example of the whole procedure (subsection 5), in order to clarify the most critical steps of the algorithm. In the Results we evaluate the cost criterion performances (subsection 1) and apply the indexes *C*, *R* and *S* to several kinds of simulated activities (subsection 2) and to real data (subsection 3)

In subsections 2 and 3 we also compare the results obtained on simulations and on real data and the information provided by constant time bin and conditional sampling. In the Discussion we briefly discuss advantages and drawbacks of the techniques developed.

## Materials and Methods

### The sampling procedure

**Figure 1:**
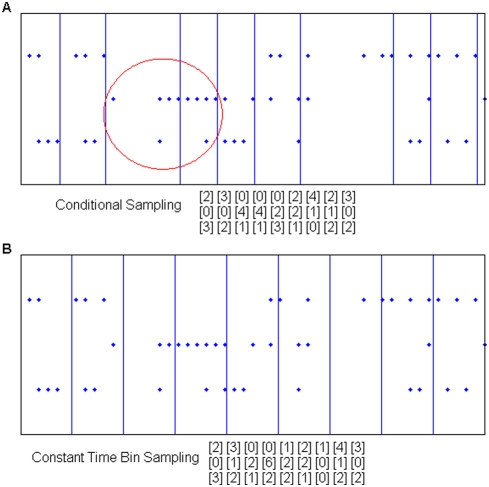
Two different sampling procedures. A) Conditional sampling. This procedure may provide adaptive time windows and detect as equal time-warped patterns with the same spatial distributions over the spiking sources. In this case note that by setting *k* = 8 three equal *k*-sequences are detected, as evidenced by the red ellipse). B) Constant time bin sampling applied to the same pattern. This is the most classical sampling procedure and is draw for comparison.

Given *k* spikes and *N* sources, we define a *k*-sequence as an *N*-dimensional vector whose values, on each dimension, are the number of spikes counted on each source, the sum over all dimensions being *k*. By fixing *k* and *N* it is possible to extract a finite set of *k*-sequences from each multisite recording (Fig. 1A).

Consider a set of *N* spiking sources and let *x_1_*, …, *x_N_* be the number of spikes emitted by them in a variable time period. Then setting a constant sum *Σ_n_ x_n_* = *k* and collecting sets of *k*-sequences, we sample from the conditional probability distribution
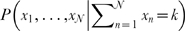

*K*-sequences are sampled along the multichannel recording raster-plot on adjacent non-overlapping windows of variable duration (Fig. 1A). Conditional sampling does not rely on an “external” clock-the time of recording, but simply on the spike occurrences across the multiple emitting sources. This peculiar property makes this procedure, to a great extent, insensitive to the common mode frequency modulations that affect all the sampled spiking sources in the same way. A simple example of this property is provided in Document S1.

Unlike the classical constant time-bin sampling (reported in Fig. 1B for comparison), conditional sampling is robust relative to the time-warp, enabling us to consider patterns of different duration and equal spatial distribution just like a same pattern (see red circled samples in Fig. 1A). In analogy with *k*-sequences, we also define *t*-sequences as samples collected by using the classical constant time bin sampling procedure. In this context *t* represents the number of time-units *Δt*, being this term the largest time interval in which a source can emit at most one spike (typically *Δt* = 1 ms). More generally, when we will refer to both *k*-sequences and *t*-sequences, we will simply write sequences.

### The clustering procedure

The clustering procedure follows a bisecting divisive partitioning algorithm. At each iteration, the cluster bisection is obtained by applying in cascade the Principal Direction Divisive Partitioning (PDDP, see [22]) and the Bisecting K-means (BK) algorithms.

Iterative separations are stopped when the dimension of each cluster is smaller than a predefined threshold *D_max_*. The cluster dimension *D_j_* of a *j^th^* cluster, is computed as
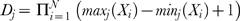
and *X_i_* is the set of values of the *i^th^* dimension associated with the sequences belonging to the *j^th^* cluster. For each cluster *j*, *D_i_* can be interpreted as the number of all its possible distinct sequences and *D_max_* as the maximum number for any cluster.

At the first step the whole dataset is bisected. Then, before any successive bisection, clusters are ranked by their dimension *D* and bisection is performed on the largest cluster. This criterion allows the obtaining of clusters which are roughly comparable in their cluster dimension.

Once we have chosen the cluster to bisect, we apply PDDP with the following steps. First, we subtract to each *i^th^* dimension (of the chosen cluster) its mean value. Then, we compute the covariance matrix of the k-sequences belonging to the chosen cluster and we extract the eigenvector ***v*** associated with the largest eigenvalue. Finally, given two subclusters *G_1_* and *G_2_*, we assign each sequence ***x*** to one of them by evaluating the score *A =* ***x*** *^T^* ***v***. When *A* is larger than 0, we assign ***x*** to *G_1_*. Otherwise we assign x to *G_2_*. After preliminary bisection by PDDP we compute the centroids ***w_1_*** and ***w_2_*** on, respectively, *G_1_* and *G_2_*, and use them to initialize the BK algorithm.

The BK algorithm is iterative and is composed of two steps. First, each item is assigned to the nearest centroid. Then, the centroids are recalculated on the base of the last assignment stage. *K*-means always converge so that after a number of iterations centroids no longer change their positions. For a more detailed treatment of PDDP and BK refer to [22]–[24].

As shown by Savaresi and Boley [23], the PDDP algorithm provides a wise centroid initialization while the BK algorithm refines the partitioning. The distinct clusters obtained by the clustering procedure constitute the sequence classes. Decreasing the values of *D_max_* we obtain a more detailed description of the dataset, with a larger number of lower dimension sequence classes. However, too small *D_max_* values may lead to overfitted descriptions failing to capture the salient properties of the noisy discharge patterns. A solution to this problem is proposed in the following paragraph.

### *D_max_* selection and essential pattern extraction

In the present subsection and in the next we propose a general evaluation method applicable to both *k*-sequences and *t*-sequences. Thus, although we will always refer to *k* and *k*-sequences, the same analysis could be applied to *t*-sequences simply by *k* to *t* label substitution.

To choose the best *D_max_* value we associate each *D_max_* with a cost. The cost is evaluated taking into account the encoding length (in bits) needed to represent a selected model and, using a model-dependent encoding, the data. Being *c_min_(k,D_max_)* the smallest cost associated with a selected *D_max_* value, we define
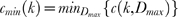
as the smallest cost, given *k*, of a *k*-sequence dataset.

The encoding algorithm relies on a re-elaboration of a scheme proposed by Willems [25] and is described in detail in Document S1. This scheme has a simple and direct interpretation in terms of information theory, being straight on implementable as a true compression algorithm.

In brief, the *k*-sequence classes are ordered in a database by the number of their occurrences. The database represents the model. Among the possible subsets constituted by the first *M* classes, the one accomplishing the shortest encoding length is selected. To exploit the model knowledge about an *i^th^* class we need first *2Nlog(k+1)* bits to store the class in the model and then *log(i)+log(D_max_)* bits at each occurrence of a *k*-sequence belonging to that class (for details see Document S1). Because *log(i)* is an increasing function of the class position in the database, classes occurring at high frequency have a smaller cost than rare ones. Not all classes are necessarily stored in the model. Some of them may not occur enough times to be conveniently stored. The classes contained in the model constitute the essential patterns we are looking for to describe the most regular part of our finite dataset.

The total cost of encoding the data without the database is
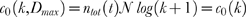
and *n_tot_(k)* is the overall number of *k*-sequences. *Nlog(k+1)* represents an upper limit for the number of possible *k*-sequences not accounting for the fact that the spike sum over all dimensions is *k*. This is done for the sake of generality. In fact, in the present form, the cost criterion can be used with any kind of sampling procedure (like constant time bin sampling). This approach is equivalent to the use of a common encoder without any *a priori* knowledge on the sampling procedure used.

To select the best model we start to test the first class by computing
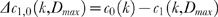
and, iteratively, test an increasing class number. At each iteration *i*, we evaluate the differential cost *Δc_i,i−1_(k,D_max_)* of adding a new *i^th^* class. Such cost is divided into three components
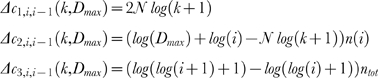
and *n(i)* indicates the number of the *i^th^* class occurrences in the dataset.

*Δc_1,i,i−1_(k,D_max_)* represents the differential database cost, that is the number of bits we need to store a class in the database. *Δc_2,i,i−1_(k,D_max_)* represents the gain, achievable by encoding *n(i) k*-sequences once stored the *i^th^* class in the database. *Δc_3,i,i−1_(k,D_max_)* represents the differential flag cost (see Document S1). The database cost constitutes a regret term for too short datasets and fades away for long sequences, while the flag cost increases linearly with the length of the dataset thus constituting a constant regret term for the number of classes.

For increasing values of *i*, the *i^th^* class will be accepted if the differential cost *Δc_i,i−1_(k,D_max_)* is negative.

Iterations are stopped when this is not verified. The *i^th^* class is rejected and, setting *M = i−1*, *c_min_(k,D_max_) = c_M_(k,D_max_)*.

The asymptotic upper bound for the average cost of a *k*-sequence is (for details see Document S1)
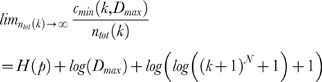
where *p* is the probability distribution of the class occurrence (all classes, not only the essentials), *H(p)* its entropy and *log(log((k+1)^N^+1)+1)* an upper limit for the flag cost *log(log(M+1)+1)*.

### Characterization of essential patterns

The application of the cost criterion described in the previous paragraph enables us to extract, for any *k*, distinct classes of essential patterns. Such patterns are associated with the *D_max_* value for which *c_min_(k,D_max_) = c_min_(k)*.

To characterize the ECs we use three measures: *C(k)*, *S(k)* and *R(k)*.

*C(k)* stands for complexity, is a general descriptor of a *k*-sequence dataset and is given by the ratio
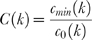

*S(k)* stands for segregation and is computed as follows
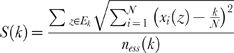
being *x_i_(z)* the *i^th^* dimension of the *z^th^ k*-sequence in the dataset and, respectively, *E_k_* and *n_ess_(k)* the set of indexes and the number of *k*-sequences belonging to ECs. *S(k)* enables us to discriminate *k*-sequences where spikes are significantly segregated in some subset of sources, from *k*-sequences where spikes are homogeneously distributed among them.

Finally, *R(k)* (reiteracy) concerns the way successive *k*-sequences shift among different classes. By the evaluation of *R(k)* we can detect the presence of stable patterns. Namely, given a symbol string associated with a multichannel recording, we estimate the probability *P_R_(k)* that successive *k*-sequences belong to the same EC
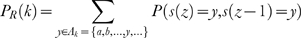
where *z* is the *z^th^* symbol of a string associated with the *z^th^ k*-sequence of the dataset and *A_k_* is the set of symbols representing all the distinct ECs (only the essential, not all classes). If *P_R_(k)* is significantly higher than expected by chance, reiteracy is detected and we set *R(k) = 1*, otherwise *R(k) = 0*.

An approximated *P_R0_(k)* distribution under the null hypothesis is obtained by *N_S_* shuffles on the original symbol sequence. We call *N_<_* the number of estimates for which *P_R0_(k)<P_R_(k)*. The null hypothesis is rejected at level *α* if *α>(1−N_<_/N_S_)*.

The indexes *C*, *R* and *S* may be meaningfully applied to both *k*-sequences and *t*-sequences. In case of *k*-sequences, *C(k)* represents the spatial complexity connected to the number and the diversity of all the possible configurations collected from a multichannel recording regardless of their duration. *R(k)*, the shift indicator between two different *k*-sequence classes, determines the switching properties of the system. Positive reiteracy on *k*-sequences may be due to small reverberant circuits, to intrinsic single neuron spiking dynamics or, more generally, to any stable differential activity that emerges over common mode modulations of the considered channels. Finally, *S(k)* represents the average euclidean distance from the homogeneous sequence, i.e. that sequence where all spikes are equally distributed among the sources.

The significance of *C*,*R* and *S*, when dealing with t-sequences (respectively *C(t)*,*R(t)* and *S(t)*) is less unambiguous. In fact, while *C(k)*,*R(k)* and *S(k)* values simply evaluate spatial regularities, *C(t)*, *R(t)* and *S(t)* values mix spatial and temporal information. Thus, a *t*-sequence may have a low *C(t)* because of either stereotyped spatial configurations or regular frequency modulations (or both). The same remark holds true for *R(t)* and *S(t)*. Although positive reiteracy may be due to stable spatial patterns and significant segregation to a sparse source recruitment, both *R(t)* and *S(t)* may be substantially affected by frequency modulations slower than the sampling frequency.

### An Explanatory Example

To gain a better understanding of the whole procedure let's consider the example data shown in Fig. 1A. After conditional sampling, the associated *k*-sequence set, given *k* = 5, is displayed in Fig. 1A (bottom). As this dataset is a bit too short for our analysis we build a larger one simply by repeating six times each item. Then, we add some noise deleting one spike from each *k*-sequence and reassigning it randomly to one of the 3 channels of the same *k*-sequence. The resulting set is

2 2 1 1 1 2 4 2 3 3 3 0 0 1

0 1 3 4 2 2 1 0 0 0 1 3 3 1

3 2 1 0 2 1 0 3 2 2 1 2 2 3

2 4 1 3 2 3 0 0 0 1 3 1 2 2

2 1 2 0 0 0 3 4 2 3 2 2 1 0

1 0 2 2 3 2 2 1 3 1 0 2 2 3

2 1 1 1 2 3 3 3 1 3 0 0 0 3

1 3 3 1 2 2 0 1 1 1 3 4 3 1

2 1 1 3 1 0 2 1 3 1 2 1 2 1

3 2 2 2 2 0 1 1 2 4 2 3

2 2 1 0 1 4 4 2 1 0 2 1

0 1 2 3 2 1 0 2 2 1 1 1

We apply the clustering algorithm with three different values of *D_max_*, respectively 4,12,48,75.

By associating a symbol with each distinct cluster we get the following strings:

*abcdefgahhillafgehahldecmebabccafmhiaildlimfbabddebgfi*

*aabbccdaaadbbacdcaaabbcbdcaaabbacdadadbbbddcaaabbcadcd*

*aabbbbaaaaabbababaaabbbbabaaabbabaaaaabbbaabaaabbbaaba*

*aabbbbaaaaabbababaaabbbbabaaabbabaaaaabbbaabaaabbbaaba*

Because *D_max_* value is critical, we select the best description by using the evaluation criterion described in paragraph 3 and in Document S1.

The description associated with *D_max_* = 12 is selected as the most synthetic, by our criterion, being the complexity *C(5)* = 0.95.

The dimension of the clusters *a*,*b*,*c*,*d* are respectively 12,12,9,12. The cost *c_0_(5)* is 418.76 bits, while *c_min_(5)* = 398.84 bits, being the differential database cost *Δc_1,3,0_(5,12)* = *2MNlog(k+1)* = 2*3*3*log(6) = 46.53 bits, the differential gain *Δc_2,3,0_* = Σ*_i_(Nlog(k+1)−log(i)−log(D_max_))n(i)* = −152.34 bits and the differential flag cost *Δc_3,3,0(_5,12)* = *t(k)log(log(M+1)+1)* = 85.59 bits.

*K*-sequence classes *a*,*b*,*c* are defined by our criterion as essential, while *d* does not occur enough times. Writing ‘*-*’ for non-ECs the final string is

*aabb--daaadbba-d-aaabb-bd-aaabba-dadadbbbdd-aaabb-ad-d*

On this string we finally compute, on ECs, reiteracy and segregation (*S* = 2.24). To evaluate *R(5)*, we calculate *P_R_* = 0.31 on the original final string, then we shuffle the final string *N_S_* = 10000 times in order to obtain *P_R0_* (Fig. S1). Having set *α* = 0.05, and being *(1−N_<_/N_S_)* = 0.046 we conclude that reiteracy is significant (*R* = 1) at level 0.05 (but not at level 0.01).

We can repeat the same procedure with constant time bin sampling. To obtain a reasonable dataset we replicate 6 times the data in Fig. 1B and add noise as in the previous case. The resulting set is

2 2 1 0 2 2 0 4 3 2 3 0 0 1

0 2 1 5 2 1 1 1 0 1 2 3 6 1

3 2 1 3 1 2 0 2 2 2 1 0 2 3

2 0 4 4 1 3 1 0 1 1 0 4 4 2

1 0 0 0 1 1 1 5 3 3 1 0 0 1

2 1 3 1 3 2 1 3 1 1 0 3 1 2

2 0 0 1 2 0 4 3 2 2 0 1 1 2

2 1 5 1 3 1 0 1 1 2 3 6 3 3

2 2 3 3 0 0 3 1 2 2 0 1 1 0

1 3 4 1 3 0 0 0 2 0 4 2

0 2 0 0 0 3 5 2 3 1 1 0

0 2 1 4 3 0 3 3 0 0 2 3

Then we apply the whole algorithm again. With *D_max_* = 4,12,48,75 we obtain the following strings:

*abcdbbefgbhjkiblgmifcdjjegmbbndioeghbbjkjolhmagjdnoefa*

*abcdbbefgbfhdabcgfafcdhhegfbbedahegfbbhdhhcffaghdehefa*

*aaabaacddadcbaaaddadabcccddaacbaccddaacbccaddadcbcccda*

*aaabaabaaaabbaaaaaaaabbbbaaaabbabbaaaabbbbaaaaabbbbbaa*

Because of the larger number of possible *t*-sequences in respect to *k*-sequences, we expect the optimal *D_max_* value to increase. In fact the final string is associated with *D_max_* = 75

*aaa-aa-aaaa--aaaaaaaa----aaaa--a--aaaa----aaaaa-----aa*

being the dimension of the class *a* equal to 75 and the values of C,S equal to respectively 0.9966, 1.6016. To use the cost criterion described, we set *k* at the largest value collected on a single channel during constant time bin sampling (*k* = 6).

Being *(1−N_<_/N_S_)* = 0.0406 we conclude that reiteracy is significant at level *α* = 0.05.

### Cost criterion performances

In this paragraph we analyze the relation between Entropy (*H*) and our cost criterion evaluation. We test the cost criterion on two different sources. We then compute *c_min_(k_max_)* as a function of the message length *n_tot_*. A source is composed of a regular and a random part. The regular part is constituted by few sequences that are repeated a number of times. The random part is constituted by random sequences generated by *N* independent extractions from a homogeneous distribution: namely, all integers ranging from 0 to *k_max_* have probability *1/(k_max_+1)* of occurrence.

Each message is composed of *r n_tot_* and *(1−r) n_tot_* sequences belonging, respectively, to the regular and to the random part.

We define *n_r_* the number of distinct sequences of the regular part. Each of them occurs *rn_tot_/n_r_* times in a message.

**Figure 2:**
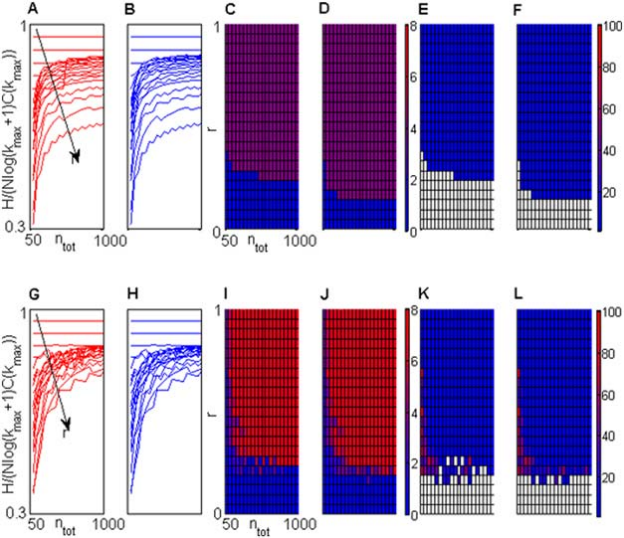
Cost criterion performances. Upper row (plots A,B,C,D,E,F) related to the first source. *k_max_* = 10, *N* = 5. A) The function *H/(C(k_max_)Nlog(k_max_+1))* is estimated, as a function of *n_tot_*, at different *r* values ranging from 0 to 1. B) The same as in A). Here the cost criterion is modified to start evaluation from *Δc_2,1_* instead of *Δc_1,0_*. C,D) *M* values estimated with the original (C) and the modified (D) criterion. E,F) *D_max_* estimated with the original (C) and the modified (D) criterion. Lower row (plots G,H,I,J,K,L) related to the second source (*k_max_* = 10, *N* = 5).

If *N* and *k_max_* are not too small
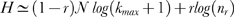
For an infinitely long message of *n_tot_* sequences, *H* represents the smallest possible average description length of a sequence. The asymptotic evaluation of our cost criterion, *c_min_*(*k_max_*), in Document S1, provides a significantly less parsimonious description. This holds true also for finite length messages, as can be observed in Fig. 2A,B,G,H, where the average values of *H/(Nlog(k_max_+1)C(k))* are plotted as a function of *n_tot_*. The difference between entropy and *c_min_*(*k_max_*) descreases as *n_tot_* increases. Conversely, it strongly increases as a function of *r*.

The more regular the message, the worse *c_min_*(*k_max_*) approximates *H*. The differential database cost *Δc_1,M,0_ = 2Nlog(k_max_+1)*, accounting for the lowest *H/(Nlog(k+1)C(k))* values at low *n_tot_*, fades away for increasing values of this parameter (see Methods section). The differential flag cost *Δc_3,M,0_* is independent of message length and accounts for the non-optimality at large *n_tot_* values. *Δc_3,M,0_* plays a key role in the choice of the EC number, typically leading to a conservative selection that prevents from overfitting. The contribution of *Δc_3,M,0_* could be reduced by modifying and expanding the flag and the database structures, in order to use a single flag to encode several sequences. This possibility, under current experimental check, can effectively reduce *Δc_3,M,0_* and the number of flags, and will be presented in a forthcoming paper. The differential flag cost *Δc_3,M,0_* is sometimes too large and may impair the detection of regular patterns that are significantly present in the message. To avoid the problem, the cost criterion can be evaluated starting from *M* = 1 instead than from *M* = 0. Results obtained with this modification are drawn in Fig. 2B,D,K,H,J,L while those relative to the original algorithm are presented in Fig. 2A,C,E,G,I,K. The values of *M* and *D_max_* are reported, respectively, in Fig. 2C,D,I,J and in Fig. 2E,F,K,L.

The regular part of the first source (Fig. 2A–F) is composed of the following sequences

1 10 5 1

3 5 5 2

10 0 5 3

10 2 5 10

2 9 6 10

having set *k_max_* = 10. For short *r* or *n_tot_* values no ECs are detected from our algorithm (white region in Fig. 2E,F). Instead, increasing these parameters, the algorithm always extracts four distinct ECs with *D_max_* = 1(blue region in Fig. 2E,F). The sharp transition between 0 and 4 ECs can be expected because the regular sequences we introduced are really different from each other.

The following sequences

1 0 1 0 2 0 0 2

3 4 4 4 3 3 4 4

10 10 9 10 10 10 10 10

10 9 10 10 10 10 10 9

2 3 2 2 1 3 2 1

are used to generate the regular part of the second source (Fig. 2G–L). No ECs are detected for low *r* or *n_tot_* values (white region in Fig. 2K,L). With slight increments of these parameters, a single EC is detected containing 7 or 8 distinct sequences of the regular part (violet region in Fig. 2I,J and red region in Fig. 2K,L). When the EC contains all 8 sequences *D_max_* = 72. In comparison with those of the first source, these sequences are very similar to each other. Accordingly, when the number of their occurrence is not too high, the algorithm detects them as different noisy versions of a unique essential pattern. For further increments of *n_tot_* or *r*, our algorithm returns, as ECs, 7 of the 8 sequences composing the regular part of the message (red region in Fig. 2I,J and blue region in Fig. 2K,L). As expected, when the repetition of single sequences becomes significant, the regular sequences cannot be seen as a single noisy pattern. In fact, if it is true, the occurrence of the distinct 72 sequences represented in the EC should roughly follow a homogeneous distribution. This is not the case because only 8 out of 72 sequences occur with high probability (*r/8*) while the remaining ones have probability *(1−r)/11^5^*.

A number of other sources have been tested, by varying *k_max_* and *N*, with matching results. In general, the non optimality of the cost criterion leads to conservative choices about the number of the ECs. Moreover, by slightly modifying the cost criterion, the selection of ECs becomes more inclusive. The algorithm typically achieves good performances in the separation of regular components from noise. These include well-tuned generalization capabilities to avoid noise-induced multiplication of the ECs for messages that are too short.

## Results

### Simulations

We used four different simulation groups to validate the algorithm.

1. We generated independent geometric processes with the parameter *p_a_ = [p_a1_ … p_aN_]* for a wide range of *N* sources and spiking frequencies *f_a_ = p_a_\Δt*.
2. We generated *M* independent geometric processes with the parameter *p_a_*, given *M≤N*. We called these events *activation processes* (Fig. 2A). At each event of activation, processes were assigned to a random subset of *N\M* sources (Fig. 2B). Then, for a period of *T* time-bins (the length of the activation period), the activated sources were driven by independent geometric processes with parameter *p_b_ = f_b_ Δt*.
3. We used the same strategy as simulation group 2, but we assigned the *M* activation processes to *M* predefined subsets of *N\M* sources.
4. All predefined *M* subsets were driven by a common trigger following a geometric process with parameter *p_a_*. When an event took place all subsets were activated at successive lags of *T* time-bins.

Activation processes embody the strong non-stationarity of neuronal activity typical both of conscious and unconscious states. In particular, they closely mimic the transition between ‘up’ and ‘down’ states recorded both intracellularly and extracellularly during recordings of ongoing activity in sleep or anesthesia [26]. The term ‘up’ states commonly indicates a depolarized state of intracellular potential typically constituting the substrate for high frequency discharges. Conversely, the term ‘down’ states indicates a hyperpolarized state of intracellular potential leading the cell firing activity to very low frequency regimes.

**Figure 3:**
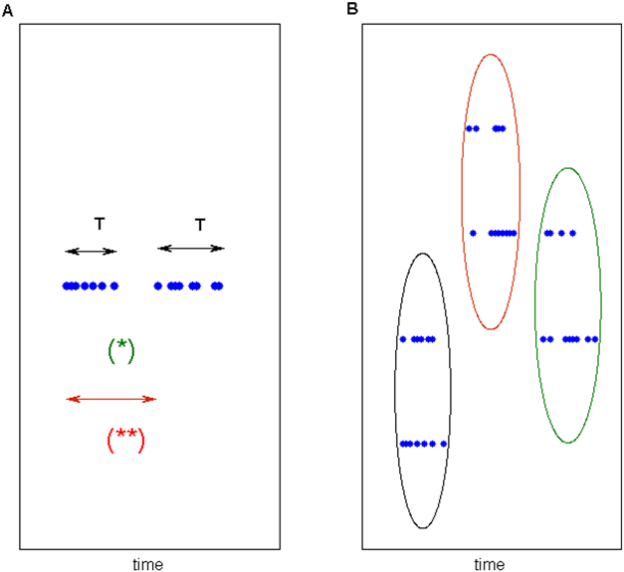
Structure of simulations 2,3,4. A) Structure of a single simulated source. Spikes are displayed as blue points. (*) Represent an inter-spike interval during an active state, having set at *f_b_* the mean frequency during the active periods. (**) Represent a period between two successive activation given *f_a_* the mean frequency of activation occurrence. B) Given N = 4 channels, we can observe three successive activations of overlapping subsets of N/M = 2 channels.

Homogeneous poisson-like activity is simulated by group 1 (Fig. 3A). Multisite activation processes can randomly distribute over the spiking sources (like in group 2, Fig. 3B), repeat on stereotyped subsets of sources (group 3, Fig. 3C) and even exhibit precise serial patterns among the different subsets (group 4, Fig. 3D). Such serial patterns were effectively observed in hippocampal place cells of rats [19] in recordings performed both during spatially constrained tasks and, soon after that, during sleep. Task-related serial patterns expressed during asleep could be time-compressed up to a factor of 20.

Constant time bin sampling was obtained by dividing each simulation into constant periods containing, on average, *k* spikes. The x axes in plots H–J represent these progressively augmenting periods called, for brevity, *<k>*.

When applied to *k*-sequences, the evaluation criterion, described in subsection 2 of Methods, works quite well for large *k* values, typically for *k*>5, while fpr *k*< = 5, the flag cost *Δc_3,1,0_* is often much higher than the gain *Δc_1,M,0_* and the overall minimum cost is *c_0_(k)*. In these cases, as suggested in Methods, *R(k)* and *S(k)* have been computed by skipping *Δc_1,0_(k)*.

**Figure 4:**
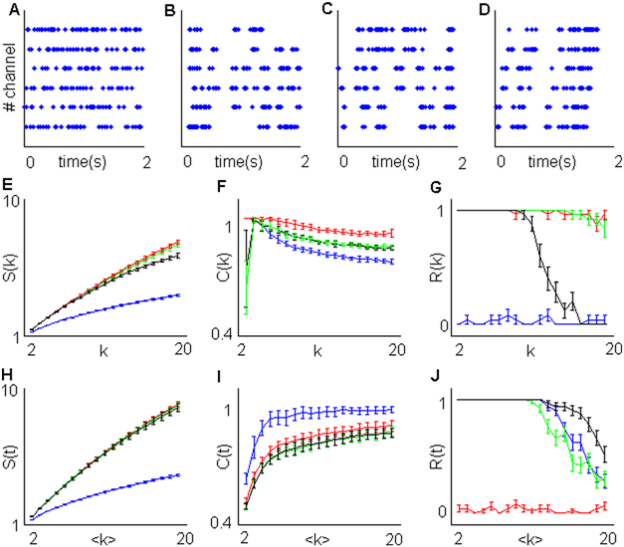
Some typical results from simulations. Time-bin resolution *Δ*t was set at 1 ms while the total number of spikes on each simulation was 5000. A) Sample plot of a group 1 simulation given *N* = 6, *f_a_* = 2 Hz. B,C,D) Sample plot of simulation belonging to group 2,3,4. We set N = 6, *f_a_* = 5 Hz, *f_b_* = 50 Hz, M = 3, T = 50 ms. E,F,G) Estimation of *S(k)* (E),*R(k)* (F) and *C(k)* (G). H,J,K) Estimation of *S(t)* (H),*R(t)* (J) and *C(t)* (K). Blue, red gree and black lines respectively represent group 1,2,3,4.

**Figure 5:**
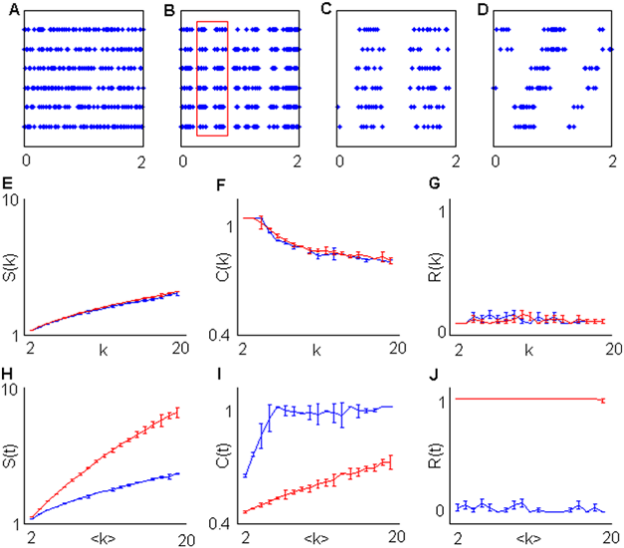
Rejection of common mode modulation in simulations. Time-bin resolution *Δ*t was set at 1 ms while the total number of spikes on each simulation was 5000. A) Sample plot of a group 1 simulation given *N* = 6, *f_a_* = 2 Hz. B) Sample plot of simulation belonging to group 2. We set *N* = 6, *f_a_* = 5 Hz, *f_b_* = 50 Hz, *M* = 1, *T* = 50 ms. C) Magnification of the plot B indicated by the red rectangle D) Sample plot from a group 3 simulation where we set *M* = 3 (the other parameters are kept fixed). E,F,G) Estimation of *S(k)* (E),*R(k)* (F) and *C(k)* (G). H,J,K) Estimation of *S(t)* (H),*R(t)* (J) and *C(t)* (K). Blue and red lines respectively represent group 1,2.

A number of simulations were performed for a wide range of *f_a_*, *f_b_*, *M*, *N* values. On each simulated dataset we applied the whole algorithm flow described in Methods. Some typical outcomes are displayed for *k*-sequences in Fig. 4E–G,5E–G and for *t*-sequences in Fig. 4H–J,5H–J. The results obtained with conditional and constant time bin sampling can be similar (Fig. 4E–J) or very different (Fig. 5E–J).

These two conditions were obtained by keeping fixed *f_a_*, *f_b_*, *M*, *N* values and setting, respectively, *M* = 3 (Fig. 4) and *M* = 1 (Fig. 5).

In Fig. 4 it is easy to see how, by using *C(k)*,*R(k)* and *S(k)* and given the same values of *f_a_*, *f_b_*, *M* and *N*, the different groups 2, 3 and 4 are effectively discriminated and suitably different from the simulations belonging to group 1. The distinction between groups 2 and 3–4 and between groups 4 and 2–3 is evident respectively for mean *C(k)* and *R(k)* or *S(k)* values, while group 1 is clearly separated from the others in all the plots. In a way, also *C(t)*, *R(t)* and *S(t)* allow for a net discrimination among the different kinds of simulations.

Strong discharge pattern segregation is present in small channel subsets in simulation groups 2,3 and 4 (Fig 4B–D). Accordingly, segregation values *S(k)* and *S(t)* (Fig. 4E and 4H) in these simulations are much larger than in group 1 (Fig 4A), where spiking discharges contained in the sequences are, on average, more homogeneously distributed over all the spiking sources.

In group 2, the subsets of *N/M* = 2 sources are randomly assigned across the *N* = 6 channels, so that one among the *N!/(N−N/M)!(N/M)!* = 15 different subsets may be selected at each activation occurrence. Instead, in simulation groups 3 and 4, the subsets are predefined and only 3 non-overlapping subsets may be activated. The different spatial complexity between simulation group 2 and simulation groups 3 and 4 is reliably detected by *C(k)* and *C(t)* values (Fig. 4F,I).

Reiteracy *R(k)*, like *S(k)*, enables us to distinguish between simulation group 4 and simulation groups 2 and 3. Simulation group 1 displays negligible reiteracy (Fig. 4G,J). The non-null values are due to the decay of the significance level *α*, caused by multiple comparisons. Simulation groups 2 and 3 exhibit substantive reiteracy along the considered *k* interval. The sharp *R(k)* decay in simulation groups 4 reflects the regular transition between non-overlapping source subsets. The *k* value coupled with the decay is proportional to the product N*Tf_b_/M*, as shown by additional simulations in Fig. S2. Given that N*Tf_b_*/M is the average number of spikes occurring during a subset activation, this last result is not surprising.

Unlike *R(k)*, *R(t)* decays in simulation groups 4 is faster than in simulation groups 2 and 3. The higher reiteracy *R(t)* in simulation group 4 is due to the longer silent periods between activation occurrences.

In simulation group 1, independently from f_a_, we did not detect significant stable patterns.

In Fig. 5, having only one subset including all channels (*M* = 1), simulation groups 2–4 are equivalent. For this reason in Fig. 5 we reported only the results obtained with simulation groups 1 and 2 (Fig. 5A–C,E–J).

No reiteracy was detected with *k*-sequences in simulation group 2 (Fig. 5J). This is expected because setting *M* = 1 means that channels are all equally modulated. Sample plots from this group are reported in Fig. 5B,C. We also reported, for the sake of comparison, a sample plot from a simulation belonging to the group 4 with *M* = 3 (Fig. 5D). Note that the activation of the different channel subsets follows stereotyped serial patterns. This is not the case for simulation group 2 (Fig. 5B,C). All the channels are simply turned on and off at the same time so that no spatial reiteracy should be detected with conditional sampling. This kind of sampling, as explained in Materials and Methods and Document S1, is insensitive to most of the common mode frequency modulations.

Moreover, no significant difference between simulation groups 1 and 2 can be detected either in terms of segregation S(*k*) (Fig. 5E) or complexity C(*k*) (Fig. 5F).

Conversely, constant time bin sampling provides remarkable differences in all the measures (Fig. 5H,I,J). In particular, strong reiteracy is due to the slow frequency modulations represented by the alternation of active and silent periods across all the simulated channels.

Constant time bin sampling mixes temporal and spatial information. Constant time bin and conditional sampling provide matching results when spatial information is salient and represents a major determinant in the clustering procedure (Fig. 4). Otherwise, when the clustering is dominated by frequency modulations, these two sampling procedures can provide incoherent results (Fig. 5).

### Real data

Several recordings of ongoing activity were analyzed both in normal and neuropathic isofluorane-anaesthetized rats. Experimental methods and general results are reported in a dedicated paper (Storchi et al. Submitted) while here we just focus on three recording groups to show and discuss some typical outcomes.

**Figure 6:**
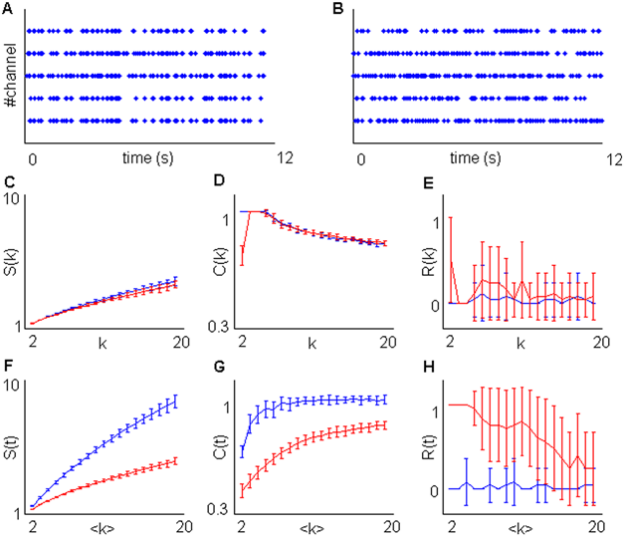
Recording from normal rat 1 (*N* = 5). A) A sample from the analyzed activity. B) Simulated group 1 activity. The mean frequency on each channel was set at the same value of the associated channel from the recorded activity. C,D,E) Estimation of *S(k)* (C),*R(k)* (D) and *C(k)* (E) from the recorded (red lines) and the simulated (blue lines) activities. Note that, although generalized oscillations are strong in all channels, the nested organization detected with *S(k)*,*C(k)* and *R(k)* is not different from the random simulated activity. F,G,H) Estimation of *S(k)* (F),*R(k)* (G) and *C(k)* (H) from the recorded (red lines) and the simulated (blue lines) activities. Note the strong reiteracy *R(t)* due to successive silent periods and compare with the negligible reiteray *R(k)*.

**Figure 7:**
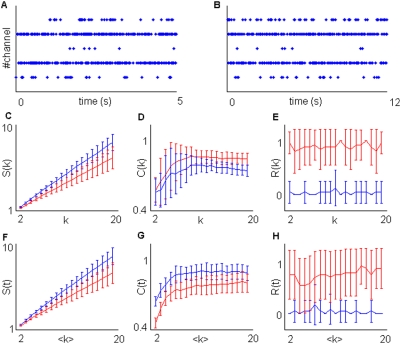
Recording from neuropathic rat (*N* = 5). A) A sample from the analyzed activity. B) Simulated group 1 activity. The mean frequency on each channel was set at the same value of the associated channel from the recorded activity. C,D,E) Estimation of *S(k)* (C),*R(k)* (D) and *C(k)* (E) from the recorded (red lines) and the simulated (blue lines) activities. F,G,H) Estimation of *S(k)* (F),*R(k)* (G) and *C(k)* (H) from the recorded (red lines) and the simulated (blue lines) activities. Discharge patterns are remarkably more stable (see *R(k)* and, to a less extent, *R(t)*) than in the normal rats 1 and 2.

**Figure 8:**
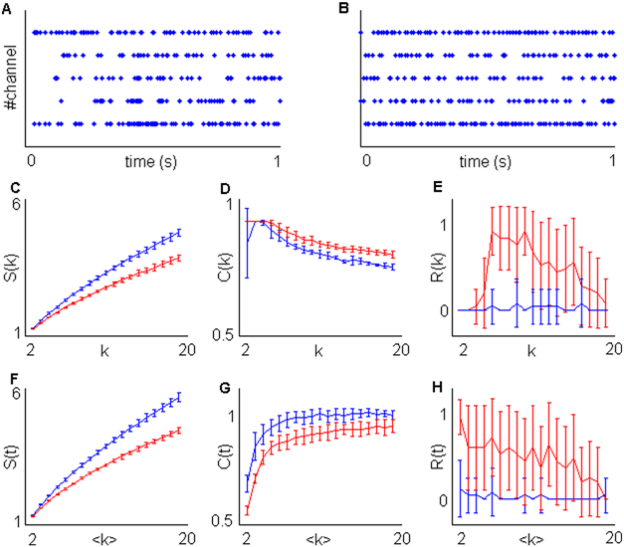
Recording from normal rat 2 (*N* = 5). A) A sample from the analyzed activity. B) Simulated group 1 activity. The mean frequency on each channel was set at the same value of the associated channel from the recorded activity. C,D,E) Estimation of *S(k)* (C),*R(k)* (D) and *C(k)* (E) from the recorded (red lines) and the simulated (blue lines) activities. F,G,H) Estimation of *S(k)* (F),*R(k)* (G) and *C(k)* (H) from the recorded (red lines) and the simulated (blue lines) activities. Recorded activity strongly differs from the random-like condition.

The recording groups 1 and 2 were performed in two normal rats while recording group 3 was obtained from a rat neuropathic model (Seltzer model, [27]), known to exhibit neuropathic-deafferentative phenomenologies. The indexes were estimated in each recording group on 25 successive recording epochs, 5000 being the total number of spikes in each epoch (see Fig. 6,7,8).

Each recording epoch was compared to a simulation belonging to the group 1 for which the parameter vector *p_a_* was set equal to the values estimated in that epoch on real data (see Simulation section).

From a rough observation, the surrogated data do not show evident differences in comparison with the real ones in recording groups 2 and 3 (Fig. 6A,B, 7A,B). This could lead us to conclude that such recordings just reflect unstructured noisy discharge patterns. However, by applying our algorithm it is easy to see how the first impression could be somewhat misleading. In fact the recorded discharge patterns exhibit significant segregation (Fig. 6C,F, 7C,F) and larger complexity (Fig. 6D,G, 7D,G) in comparison with surrogated data. Moreover, reiteracy is also significant, mostly for low *k* values in recording group 2 and over all considered *k* intervals in recording group 3 (Fig. 6E,H, 7E,H).

The *R(k)* decay for increasing values of k, as can be observed in recording group 2, may provide a measure of the mean latency of repetitive configurations. For experimental applications, the detection of the *k* value associated with the *R(k)* decay (*k_decay_*) can constitute an interesting tool. The mean *k_decay_*-sequence duration represent the average switching period among different discharge pattern configurations. An altered switching period could represent a marker for altered ongoing activity such as the one we can observe in animal models of neuropathic pain (unpublished data). More generally, spontaneously switching configurations may represent a suitable neural substrate to integrate and contextualize incoming sensory information, amplifying relevant inputs and skipping irrelevant ones. Gain modulations of neural responses driven by an internal scheduling is a well-known general computational principle that enables the performance of a variety of tasks such as attention selection or coordinate transformation [28]. The capacity to switch between different active spatial configurations is embedded in recurrent neural networks [29]. EC and reiteracy can be used in this context of recurrent networks, like small cortical networks, to investigate those switching properties *in vivo*.

While *k*-sequence and *t*-sequence processing provide comparable results in recording groups 2 and 3, remarkable differences were obtained in recording group 1. Only indexes *C(t)*,*S(t)*,*R(t)* detect significant departures from the surrogated data (Fig. 8F,G,H). In fact, while *C(k)*, *S(k)* and *R(k)* indexes significantly discriminated between recording groups 2 and 3 and the relative surrogated data, this was not the case for recording group 1 (Fig. 8C,D,E). In this recording group, the presence of generalized activations synchronized across the recording sites is clearly observable (Fig. 8A,B). Fast ‘up’ states occur in the spindle oscillation regime (conventionally in the 7–14 Hz frequency interval). The presence of well-defined spindle-like oscillations could also suggest the presence of structured spatial configurations that match frequency modulations. Again a rough observation in time domain (Fig. 8A,B) is inappropriate. Both *C(k)* and *S(k)* fail to detect non-random spatial organizations in the *k*-sequence dataset (Fig. 8C,E). Moreover, negligible reiteration is detected (Fig. 8D). The whole set of measures depicts a situation very similar to the one described by simulation group 2 with *M* = 1 (see Simulations section and compare Fig. 8E–G with Fig. 5E–G). Apparently all the sources are driven by the same process and the time-variance of the driving process is the only difference with the surrogated activity. The last result suggests that within and among single fast ‘up’ states, occurring in the spindle oscillations regime, whose onset and decay are well synchronized across the recording sites, spatial organization of discharge patterns can be negligible or absent. This is consistent with the results of Kurths and coworkers [30], who simulated a biologically plausible network whose nodes where constituted by subnetworks of interacting excitable neurons. They found that weak couplings among and within nodes reflected a complex hierarchical structure, well matched with the underlying architectural connectivity, while too strong couplings resulted in an undifferentiated generalized oscillatory activity. In this regard it is important to note how conditional sampling provides results that are independent from generalized frequency modulations. The presence of reiteracy *R(k)* provides us with additional and complementary information in respect to spiking frequency oscillations. Namely stable active subsets may be present independently of common mode firing frequency modulations.

On the whole, the properties extracted by the application of index *C(k)*,*S(k)* and *R(k)* and briefly highlighted here could lead to significant advances in the analysis of ongoing brain activity.

## Discussion

In this paper we present an original method to extract and characterize sequences with fixed numbers of spikes and distributed on multisite sources in multiple electrode recordings. Being the samples collected on the base of fixed spike counts, their occurrence is detected in a time-independent fashion *k* across the spiking sources. Time independence yields a basic advantage making insensitive to brain oscillations and distinguishes spatial dynamics of multisite discharge patterns. Starting from the basic observation that the assessment of all possible patterns is unachievable because of the diverging increase of possible configurations as a function of *k* and *N*, we embedded in our algorithm a procedure, based on clustering, to extract the most salient pattern structures that we called *essential classes* (EC). To characterize the dynamics within and among the detected classes, we introduced three simple measures (C; R; S) evaluating, respectively, the average pattern complexity, the structure of ECs and their stability in time. Several kinds of surrogate activity, ranging from random to strongly structured, were used to validate the algorithm. Its application to real data significantly revealed both random and non-random spatial structures of ongoing discharge patterns and, in several cases, their remarkable stability.

The main drawback of our conditional sampling technique emerges when spiking frequency, synchrony or the number of sources is high. In these cases, when several spiking sources discharge simultaneously next to the completion of a *k*-sequence, it could happen to collect sequences containing more than the predefined *k* spikes. For example, given *k* = 5 and *N* = 2, it is possible that, during the sampling procedure, after 4 spikes there is a simultaneous emission from the two sources, resulting in a sequence of 6 spikes. A criterion to decide which of the two spikes will go in the completing sequence and which in the following could solve this problem. However any criterion will be necessarily arbitrary. Because the number of “overloaded” *k*-sequences is negligible in both the simulations and the data analyzed, we reasonably skipped the problem, potentially more demanding with larger source numbers.

As it concerns the clustering procedure, the algorithm we used [22], [23] was selected for its wise initialization and computational efficiency. We also used the farthest-first transversal clustering algorithm [31].This has the important property of keeping the clustering cost, defined as the largest cluster radius, below a twofold value of the optimal clustering (irrespectively to the number of clusters). The results were qualitatively the same as the ones shown above. In principle, any kind of clustering algorithm could be introduced in the algorithmic flow.

The use of an encoding scheme, inspired by the work of Willems [25], proved to be quite conservative in the extraction of the EC (typically 1–20), exhibiting reasonable generalization performances and avoiding overfitting. The ability to separate repetitive and random occurring sequences and the relation between Entropy and the cost criterion length have been analyzed. The scheme we described is readily implementable as a compression algorithm. We think that, in some cases, we could achieve much better compression by modifying the flag structure. In order to reduce the flag number, a single flag could be modified to encode several blocks, increasing the compression rate of those *k*-sequence datasets with regular serial dynamics. Conceptually, our approach is not very different from the first formulation of Minimum Description Length (MDL) [32], known as the two-part MDL. In adjunct to MDL, the scheme we developed allows the separation of a regular component (composed of the ECs and all their k-sequences) from a more random one. The final description length is the shortest achievable by the scheme and is asymptotically bounded by the entropy plus a constant factor.

Several algorithms have been developed in the last decades to evaluate spontaneous and evoked activities.

The method we described is original and not straightforwardly comparable with other preexisting algorithms in terms of performance. Instead, given its substantial novelties, our algorithm can be used in addition to other algorithms in order to provide complementary information. The work of Abeles and colleagues [13], [15] deals with precisely timed repeating sequences (also called “cortical songs”) while, with conditional sampling, we skip the time dimension and focus on the order of occurrence of spike groups. Thus, when observing tonic activity regimes, our pattern characterization seemingly spatially complements the precise firing reverberation sequences of well-timed activations of the synfire chain model [33].

Some authors took into account the use of relative order of spike occurrence by assigning a distinct symbol to each source [19], [34]. Those methods rely on template matching and allow for deciding whether arbitrary, user-chosen patterns occur more often than expected by chance. Such valuable techniques are currently contributing to the discovery of unexpected spiking schemes, such as time-compressed replays of behavior-related spike sequences in cortex and hippocampus [18]–[20]. Our algorithm equally uses the relative order of spike occurrences, but, with conditional sampling and clustering, it assigns distinct symbols to different multisource configurations. The method is less detailed (we skip the precise order of occurrence of single spikes in a sequence) but provides more general information about all possible recurrent patterns. The algorithm automatically selects the ECs with no need for *a priori* knowledge of the patterns to be tested.

Conditional sampling might grant complementary spatial information independent from the domain of brain oscillations. Oscillations are a widespread and complex intermingled thread, pervading, at different temporal scales, the whole brain dynamics [35], [36]. Local Field Potentials (LFP) or Electroencephalogram (EEG) provided extensive knowledge about the oscillation bands generated, among others, in the thalamo-cortical loop, and about their neural substrate and their functional significance [7]–[9].

Recent data support an extended view of neural processing from the sole time-domain measures of oscillating brain dynamics to seemingly time-independent complementary measures as introduced by our work. A recent study highlighted, by simulation runs, the presence of the same dominant discharge patterns at significantly different oscillation frequencies staggered both in wide and in sharply peaked bands [37]. Our method, based on single unit recordings, combined with the time-frequency analysis of EEG and LFP, might provide an interesting multiscale approach aimed to join population oscillatory rhythms with multisite discharge patterns.

The characterization of essential patterns in terms of stability has a solid theoretical background in the great body of work developed to investigate the attractor dynamics of neuronal networks [38]–[41]. Attractors constitute the essential elements for memory storage and retrieval. Several maintenance mechanisms for attractors have been proposed, ranging from recurrent excitation within cell assemblies to synfire-chains and single-cell bistability.

The presence of stereotyped attractor-like configurations or motifs among the whole set of possible neural combinations is a common finding in works dealing with spatiotemporal characterization of ongoing dynamics. Yuste and coworkers [11], showed the presence of precise and repetitive patterns of discharge in somatosensory thalamocortical slices using calcium imaging. Such patterns could either arise spontaneously at the onset of ‘up’ states or could be evoked by thalamic stimulation [12]. Interestingly, spontaneous and evoked patterns were statistically indistinguishable. Accordingly, they hypothesized that spatiotemporal discharge patterns are predefined in the cortex, the thalamus simply providing a trigger signal. Ordered serial activations of specific neural subsets were also observed with single units in vivo in S1 cortex of rats [10]. The result was quite generalizable because it was obtained both in urethane and ketamine-xylazine anaesthetized and in unanaesthetized rats.

When triggered by incoming sensory stimulations or by pending tasks, the spontaneous subset activations could constitute the neural substrate for gain modulations, a widely analyzed and debated general computational principle [27].

In general the algorithm we developed, thanks to conditional sampling, to its robustness in respect to noise and to the novel cost criterion, could lead to the uncovering of unobserved general properties of spiking network dynamics. We propose its application in characterization of ongoing dynamics in diverse physiological and pathological conditions (sleep, chronic pain, visual-attentive processes, memory based rehearsal of past experiences a.s.o.) Specifically, we briefly showed how our indexes *C(k)*,*S(k)*,*R(k)* could be able to characterize altered ongoing activity in an experimental model of chronic pain.

Beyond the issues already discussed, among future applications and developments, the relation between activity and the underlying anatomical substrate represents a promising field of investigation. Several theoretical works have evidenced the fundamental role exerted by structural motifs on the emergence of variable functional motifs, on different time and spatial scales [30], [42], [43]. More recently, a paper addressing explicitly the mutual relationships between network architecture and dominant patterns of neural activity stressed how synaptic connections determine the repertoire of spatial patterns in spontaneous activity [36]. Spatial configurations identified by our method could represent a further starting point to address the problem of relation between functional and anatomical connectivity.

## Supporting Information

Document S1 In the first paragraph some examples of conditional sampling are provided. In the second the encoding scheme used to develop the cost function is described in detail. In the third an upper bound for cmin(k,Dmax) is calculated.
(0.12 MB PDF)

Figure S1 Estimated distribution of P_r0_. P_r0_ estimated with N_s_ = 10000 shuffles of the final string. The estimated value of P_r_ is represented by the blue vertical line.
(1.41 MB TIF)

Figure S2 R(k) values of a group 4 simulation. We set N = 6, M = 2, T = 100 ms, f_a_ = 0.5 Hz and f_b_ = 20(blu line), 40(red),60(green),80(black) and 100 Hz(cyan). The values M T f_b_ = 4, 8, 12, 16, 20 Hz, as expected, well reflects the position of R(k) sharp decay.
(1.46 MB TIF)
