# Supplementary material for: Extraction and Characterization of Essential Discharge Patterns from Multisite Recordings of Spiking Ongoing Activity
Source: PLoS One. 2009 Jan 28;4(1):e4299. doi: 10.1371/journal.pone.0004299 (PMC2628737; doi:10.1371/journal.pone.0004299)
Supplement: Document S1 — In the first paragraph some examples of conditional sampling are provided. In the second the encoding scheme used to develop the cost function is described in detail. In the third an upper bound for cmin(k,Dmax) is calculated. (0.12 MB PDF) [file pone.0004299.s001.pdf]

Extraction and Characterization of Essential Discharge Patterns from Multisite Recordings of Spiking Ongoing Activity

Storchi R Baselli G Liberati D Biella GEM

January 4, 2009

1 Supplemetary Material[1]

1.1 Rejection of Common Modulations

Conditional sampling, in comparison with the classical constant time-bin sampling, enables to reject, at least in most cases, the common mode modulation of the spiking frequency across different spiking sources. Here we consider a simple but significant example while a more general analysis is currently in preparation:

Consider two spiking neurons and let n_1, n_2 *the number of spikes emitted in a time period* T. *They both follow a Poisson process with parameters* $\lambda_c \lambda_1 T$ *and* $\lambda_c \lambda_2 T$ *respectively, being* λ_c *a common modulation term,* λ_1 *and* λ_2 *the independent component of their activity. Let* $n_1 + n2 = k$ *and* $P_{n_1,n_2} = P_{n_1,k-n_1}$ *the probability of observing* n_1 *and* $k - n_1$ *spikes (respectively from the first and the second neuron) in a period* T. *Then*

$$P_{n_1,k-n_1} = e^{-\lambda_c T(\lambda_1+\lambda_2)} (T\lambda_c)^k \frac{\lambda_1^{n_1} \lambda_2^{k-n_1}}{n1!(k-n_1)!}$$

Now consider the conditional probability of observing n_1 *and* $k - n_1$ *spikes in period* T *given* $n_1 + n_2 = k$

$$P_{n_1,k-n_1|n1+n2=k} = \frac{\frac{\lambda_1^{n_1} \lambda_2^{k-n_1}}{x1!(k-n_1)!}}{\sum_{n_1=0}^{k} \frac{\lambda_1^{n_1} \lambda_2^{k-n_1}}{n1!(k-n_1)!}}$$

Note that this last expression is independent from the product $T\lambda_c$, *representing the common frequency modulation affecting both neurons. This result, obtained with two spiking sources, can be easily applied to any number of poissonian sources.*

More in general, given N sources, it is valid any time the joint probability

[1]Without loss of generality and in order to get smooth curves for C(k) we will skip the roundings $\lceil . \rceil$ and $\lfloor . \rfloor$ representing the smallest or the largest integer respectively larger or smaller than real a number

$Pn_1, .., n_N$ can be written as follows

$Pn_1, .., n_N = f(\lambda_c, T, n_1, ..., n_N) = h(\lambda_c, T)g(n_1, ..., n_N)$

This means that conditional sampling is effective, at least, when the all the terms containing T and λ_c can be factorized in respect to all the terms containing $n_1, ..., n_N$.

1.2 The Encoding Scheme

The encoding scheme is based on a re-elaboration of the Willems algorithm (1). This scheme translates fixed blocks into variable length codewords representing pointers to a database. When a copy of a block to be encoded is contained in such a database the block is compressed by writing its position in the database, otherwise it is left uncompressed. Increasing position values in the database lead to increasingly long pointers. The database length is constrained by the fact that pointer encoding cannot be equal or longer than the block to encode. Each codeword is constitued by two parts: a constant length flag and a variable length part. The costant length flag signals to an eventual decoder the number of expected bits occurring before the switch to the following block. The variable length part contains the pointer to the database or the block itself. In comparison with the original scheme [1] we introduce two substantially novel elements. First, we encode the position of a block in a fixed database, instead of using a sliding buffer. Our database is determined by the clustering procedure. Second, the block we point to is not a single k-sequence but instead represents a k-sequence class. Consequently, additional bits are needed to specify exactly which k-sequence has been encoded among all the possible k-sequences of that class.
Each k-sequence class in the database is represented by two elements. The first one, called root (**r**), is a k-sequence with the value of each dimension equal to the smallest number of spikes contained in a sequence of that class. The second one, called error (**e**), is a sequence with the value of each dimension equal to the largest number of spikes contained in a k-sequence of that class. The root and the error, as all k-sequences, are represented by $Nlog(k+1)$ bits. Given the **r** and **e** it is quite simple to create a recontruction algorithm to provide an indexed generation of all the possible k-sequences in the class . When both encoder and decoder are equipped with the same reconstruction algorithm, a k-sequence can be simply expressed by writing the position of its class in the database. Its index is the outcome of the reconstruction algorithm. For example let the root and the error be

r e
5 0
3 2
8 1

Note that in this case $D = 6$. Feeding with the above numbers the reconstruction algorithm we will get

5 5 **5** 5 5 5

flag	position
00	λ
01	0
01	1
10	00
10	01
10	10
10	11
11	uncompressed

Table 1: Encoding scheme

3 4 **5** 3 4 5
8 8 **8** 9 9 9

So that to encode the sequence [5 5 8], which is the 3th outcome of the given reconstruction algorithm, we will write the constant length flag, followed by the root position and by the index 3. In general, given M the number of clusters, an encoded k-sequences dataset will have the following structure:

$$\{\langle root_1\rangle\langle err_1\rangle...\langle root_M\rangle\langle err_M\rangle...\langle flag_i\rangle\langle kseq_i\rangle...\langle flag_j\rangle\langle pos_j\rangle\langle ind_j\rangle...\}$$

being $\langle flag_i\rangle\langle kseq_i\rangle$ an uncompressed k-sequence and $\langle flag_j\rangle\langle pos_j\rangle\langle ind_j\rangle$ a compressed one encoded by the position of the root in the database ($\langle pos_j\rangle$) and the index of the k-sequence ($\langle ind_j\rangle$) as generated by the reconstruction algorithm.
The flag length can be determined considering that we need a distinct flag any time the class position in the database must be encoded with a different number of bits. Moreover, an additional flag is required to signal that a k-sequence will be left uncompressed. For example if we had 7 clusters we will use the encoding scheme in Table 1 where the flag 00, indicating the first position in the database, requires no additional bits (λ) and the the flag 11 indicates that the k-sequence will be written uncompressed.

In general, given M classes we will need $log(log(M+1)+1)$ bits of flag. The presence of flags is fundamental for decodability to obtain a prefix code in a data compression context and, in our context, is functional as an additional regret to avoid overfitting.

1.3 An upper bound for $\mathbf{c}_{min}(k, D_{max})$

We first introduce two simple observations that will be used for the analysis.

OBS1: Let the discrete distribution $\mathbf{p} = \{p_1, ..., p_M\}$, where $p_m \geq p_{m+1}$ for any $m = 1, ..., (M-1)$, and $\mathbf{m}^{'} = \{\frac{1}{p_1}, ..., \frac{1}{p_M}\}$. Then, for any $m = 1, ..., M$, $m^{'}(m) \geq m$.

OBS2: Let a set of k-sequences w and its distribution $\mathbf{q} = \{q_1, ..., q_W\}$. Let *c(w)* a (clustering) function on this set and $\mathbf{p} = \{p_1, ..., p_C\}$ its distribution. Then, by decomposability of entropy, $H(\mathbf{p}) \leq H(\mathbf{q})$.

Let *L(m)* the cost of a k-sequence belonging to the mth class, M the number of k-sequence classes associated to the shortest encoding and $M_{tot} \geq M$ the total number of classes detected as a function of D_{max}. Then

$L(m) = log(m) + log(D_{max}) + log(log(M+1)+1)$
for $m \leq M$
$L(m) = Nlog(k+1) + log(log(M+1)+1)$ otherwise

For easy of notation we define

$\alpha(i) = \frac{i2Nlog(k+1)}{t(k)} + log(log(i+1)+1)$

Then the average cost $\frac{c_{min}(k,D_{max})}{t(k)}$ of a k-sequence dataset is given by

$\frac{c_{min}(k,D_{max})}{t(k)} = \sum_{m=1}^{M_{tot}} p_m L(m) + \frac{2MNlog(k+1)}{t(k)}$
$\leq \sum_{m=1}^{M_{tot}} p_m(log(m) + log(D_{max})) + \alpha(M_{tot}) \longrightarrow \{OBS1\}$
$\leq log(D_{max}) + \sum_{m=1}^{M_{tot}} p_m log(\frac{1}{p_m}) + \alpha(M_{tot})$
$\leq log(D_{max}) + H(\mathbf{p}) + \alpha((k+1)^N)$

where we exploited **OBS1**. We also know from **OBS2** that

$H(\mathbf{p}) \leq H(\mathbf{q}) \leq H(\mathbf{p}) + log(D_{max})$.

Finally, given $t(k) \rightarrow \infty$, $\frac{c_{min}(k,D_{max})}{t(k)} \leq H(\mathbf{p}) + log(D_{max}) + log(log((k+1)^N+1)+1)$.

References

[1] Willems FMJ (1989) Universal data compression and repetitions times. IEEE Transaction on Information Theory 35(1):44-53.
