# Supplementary figures and images for: Extraction and Characterization of Essential Discharge Patterns from Multisite Recordings of Spiking Ongoing Activity
Source: PLoS One. 2009 Jan 28;4(1):e4299. doi: 10.1371/journal.pone.0004299 (PMC2628737; doi:10.1371/journal.pone.0004299)

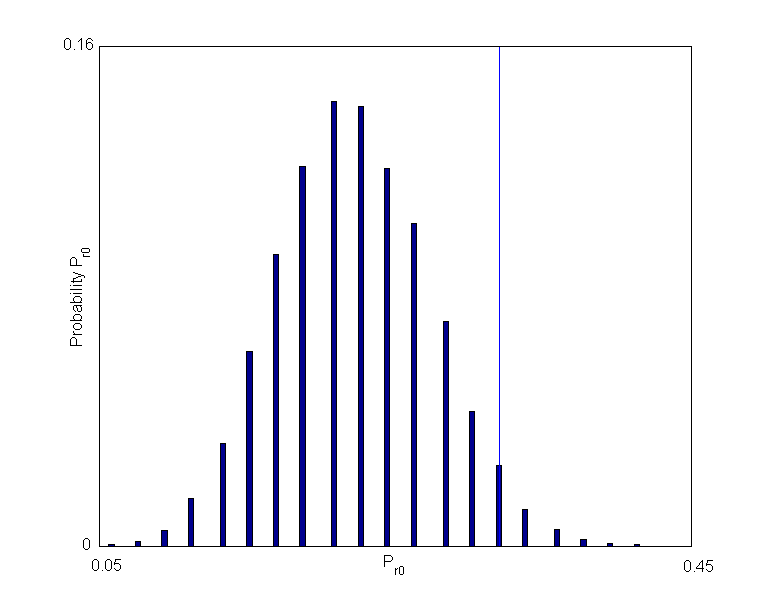

Supplement: Figure S1 — Estimated distribution of Pr0. Pr0 estimated with Ns = 10000 shuffles of the final string. The estimated value of Pr is represented by the blue vertical line. (1.41 MB TIF) [file pone.0004299.s002.tif]

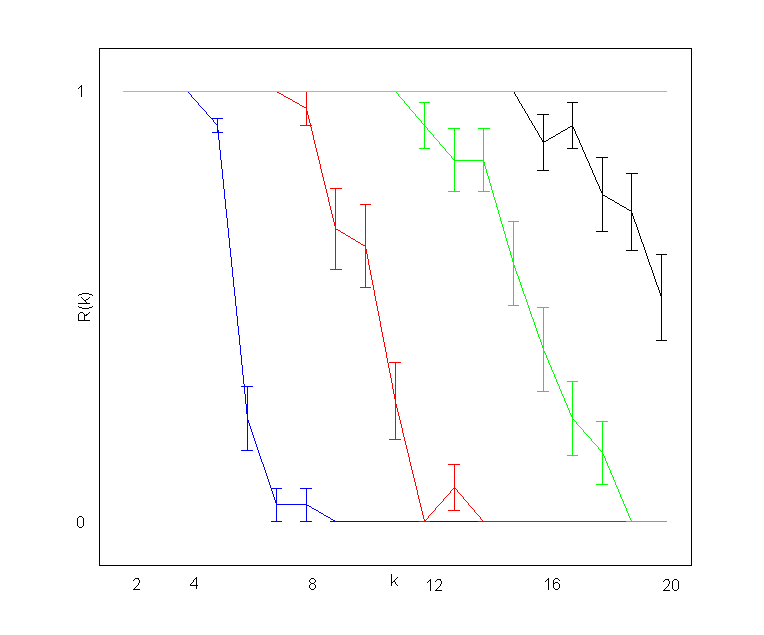

Supplement: Figure S2 — R(k) values of a group 4 simulation. We set N = 6, M = 2, T = 100 ms, fa = 0.5 Hz and fb = 20(blu line), 40(red),60(green),80(black) and 100 Hz(cyan). The values M T fb = 4, 8, 12, 16, 20 Hz, as expected, well reflects the position of R(k) sharp decay. (1.46 MB TIF) [file pone.0004299.s003.tif]
